# Adapting co-design methodology to a virtual environment: co-designing a communication intervention for adult patients in critical care

**DOI:** 10.1186/s40900-023-00514-6

**Published:** 2023-11-13

**Authors:** Laura Istanboulian, Louise Rose, Yana Yunusova, Craig Dale

**Affiliations:** 1 Daphne Cockwell School of Nursing, Toronto Metropolitan University, 288 Church St., Toronto, M5B 1Z5 Canada; 2 Michael Garron Hospital, 825 Coxwell Ave., Toronto, M4C 3E7 Canada; 3 Florence Nightingale Faculty of Nursing, Midwifery and Palliative Care, King’s College London, James Clerk Maxwell Building, 57 Waterloo Road, London, SE1 8WA UK; 4 Department of Critical Care and the Lane Fox Respiratory Unit, Guy’s and St. Thomas’ NHS Foundation Trust, Westminster Bridge Road, London, SE1 7EH UK; 5 Department of Speech Language Pathology, University of Toronto, 500 University Ave. #160, Toronto, M5G 1V7 Canada; 6 KITE: Toronto Rehabilitation Institute, University Health Network, 550 University Ave., Toronto, M5G 2A2 Canada; 7 Harvitz Brain Sciences Program, Sunnybrook Research Institute Wellness Way, Toronto, M4N 3M5 Canada; 8 Tory Trauma Program, Sunnybrook Health Sciences Centre, 2075 Bayview Ave., Toronto, M4N 3M5 Canada; 9 Lawrence S. Bloomberg Faculty of Nursing, University of Toronto, 155 College St., Toronto, M5T 1P8 Canada

**Keywords:** Co-design, Virtual, Communication, Critical care

## Abstract

**Background:**

Research co-design is recommended to reduce misalignment between researcher and end-user needs and priorities for healthcare innovation. Engagement of intensive care unit patients, clinicians, and other stakeholders in co-design has historically relied upon face-to-face meetings. Here, we report on our co-design processes for the development of a bundled intensive care unit patient communication intervention that used exclusively virtual meeting methods in response to COVID-19 pandemic social distancing restrictions.

**Methods:**

We conducted a series of virtual co-design sessions with a committee of stakeholder participants recruited from a medical-surgical intensive care unit of a community teaching hospital in Toronto, Canada. Published recommendations for co-design methods were used with exclusively virtual adaptations to improve ease of stakeholder participation as well as the quality and consistency of co-design project set-up, facilitation, and evaluation. Virtual adaptations included the use of email for distributing information, videos, and electronic evaluations as well as the use of a videoconferencing platform for synchronous meetings. We used a flexible meeting plan including asynchronous virtual methods to reduce attendance barriers for time-constrained participants.

**Results:**

Co-design participants included a patient and a family member (n = 2) and professionally diverse healthcare providers (n = 9), plus a facilitator. Overall, participants were engaged and reported a positive experience with the virtually adapted co-design process. Reported benefits included incorporation of diverse viewpoints in the communication intervention design and implementation plan. Challenges related to lack of hands-on time during development of the co-designed intervention and participant availability to meet regularly albeit virtually.

**Conclusions:**

This report describes the methods, benefits, and challenges of adapting in-person co-design methods to a virtual environment to produce a bundled communication intervention for use in the adult intensive care unit during the COVID-19 pandemic. Adapting recommended co-design methods to a virtual environment can provide further opportunities for stakeholder participation in intervention design.

**Supplementary Information:**

The online version contains supplementary material available at 10.1186/s40900-023-00514-6.

## Abstract (highlights)

**Background:**

Modifications to co-design methods were necessary during pandemic conditions but can also be useful when needing to overcome geographic and time restrictions or operating with limited resources. This report describes virtual adaptations made during the co-design of a communication intervention for patients requiring critical care. Modifications included use of a video platform for meetings, email correspondence and flexible meeting approach.

**Methods:**

Together with a critical care patient, family, and healthcare providers we developed a communication intervention and made virtual modifications to recommended co-design methods.

**Results:**

Overall, participants were engaged and described having a positive experience with the co-design process. This design also had some limitations including ongoing challenges with time availability for busy clinician participants.

**Conclusions:**

This paper may be useful for those seeking to use virtual co-design methods for intervention development and health research.

**Supplementary Information:**

The online version contains supplementary material available at 10.1186/s40900-023-00514-6.

## Background

Co-design has emerged over recent decades as a research and service improvement approach to healthcare and social change [1]. As a design-focused process that uses participatory methods, co-design responds to historical gaps in inclusive user-centered design of clinical interventions or processes [2]. Defined as the act of intentionally creating a solution with the people who will experience it, co-design prioritizes the meaningful engagement of intervention end-users (e.g., patients, family caregivers, clinicians, and managers) and researchers through study planning, design, conduct and analysis phases [1]. Co-design methods in healthcare usually begin with local stakeholders (e.g., healthcare providers [HCPs], patients, carers [e.g., family] and researchers) reflecting on their experiences of a health service or problem and working together to identify improvement priorities. This is followed by devising, and implementing changes in iterative phases, and then jointly reflecting on and celebrating achievements [1, 3]. Collaboration among stakeholders during the design stage is particularly important in co-design as it concerns the translation of user knowledge into recommendations designed to fit the practice context [4]. The outcomes of co-design include more acceptable interventions for implementation into practice and greater skills, knowledge, and positive experiences for participants [5, 6].

Co-design is theorized to balance expert researcher insight (top-down input) with real-world experience and knowledge of end-users (bottom-up input). To meet these balanced aims, it is important to foster a co-design environment that goes beyond tokenistic consultation and evaluation of end-user attitudes towards innovation. Practical strategies are necessary to merge different inputs through sustained dialogue such that both expert and end-users learn from one another. While co-design aligns with Medical Research Council guidance for the development and implementation of health interventions, there remains uncertainty about successful methods of participant engagement [1, 7]. This includes questions about sufficient end-user participation across design phases and engagement of vulnerable populations [1]. Furthermore, though co-design is widely used in healthcare, it is seldom described or evaluated in detail [1].

As co-design is also relatively new to the intensive care unit (ICU) context, research in this domain represents a vital opportunity to consider stakeholder engagement experiences and recommendations for employing this approach [1]. Furthermore, the COVID-19 pandemic placed unprecedented demands on ICU services and HCPs [8]. The shift towards social distancing and infection prevention and control (IPAC) measures presented important challenges for co-designing rapid solutions to pressing demands on ICU services. For example, IPAC measures made virtual adaptation of traditional methods of co-design including face-to-face meetings necessary in our study and others [9–11]. Exacerbation of time constraints and stress in the ICU from pandemic conditions also made the Easy, Attractive, Social, and Timely (EAST) framework of behavior change an attractive strategy to improve ICU participant engagement [12]. The EAST model recommends activities and communications that are easy to participate in or respond to (e.g., emails, short feedback forms) [12].

We conducted a mixed-method acceptability evaluation, reported elsewhere [13, 14], confirming the reported acceptability of this co-designed communication intervention for use in the ICU during IPAC conditions such as those during the COVID-19 pandemic described in this paper [15]. The current paper reports specifically on the co-design methods used, including adaptation from face-to-face engagement to a virtual delivery mode and application of EAST principles. Importantly, we also report on co-design participant experiences. Our primary objectives was to describe adaptation of our co-design processes of intervention development to virtual methods and to describe co-design participant reported experiences, including perceived benefits and challenges.

### Our project: co-designed bundled patient communication intervention for use in the ICU during and beyond the COVID-19 pandemic (COPE)

The physical and psychological sequela of communication difficulty for patients with an advanced airway in critical care has been well documented [16–20]. IPAC conditions in place (i.e., personal protective equipment [PPE], isolation, visitor restriction) during the COVID-19 pandemic exacerbated communication difficulty for ICU patients and their caregivers [14, 21, 22]. These pandemic conditions warranted the deployment of tailored solutions to address problems such as communication difficulty in critical care [23]. Gaps in the use of evidence-based communication practices suggest the need to better design and implement communication tools and provider training based on stakeholder experience including the use of co-design methods [14, 23, 24]. To accomplish this objective a series of sequential facilitated co-design sessions were conducted to produce the communication bundle prototype and implementation plan. The intervention and implementation plan are described in more detail (see Additional file 1 and Additional file 2).

### Our approach to co-design and virtual adaptations

#### Setting

The study setting is described in detail elsewhere [15]. In brief, the study was conducted in a 17-bed medical surgical ICU of a community-based hospital in Toronto, Canada. The design period occurred between May–September 2021, during which the ICU was admitting patients experiencing acute respiratory failure secondary to COVID-19 disease.

### Recruitment and participants

We used purposive and maximal variation recruitment to include professionally diverse ICU HCPs for participation in the co-design committee. We used methods such as email and poster distribution on approved unit bulletin boards, as well as during in-person staff meetings. We targeted 10–12 participants to ensure adequate representation from nursing, professions representing the ICU team, ICU leadership, the IPAC team, and at least one patient and family member. Inclusion criteria for patients and families were in ICU during the COVID-19 pandemic; age ≥ 18 years; English speaking; and access to internet and computer to support virtual meetings. All ICU staff were eligible to participate. The 11 co-design committee participants included a patient and family member dyad, a nurse educator, nurse manager, and bedside nurse, three speech language pathologists, a social worker, a respiratory therapist, and an IPAC team member. Most HCP participants worked full-time in the study setting and most gender-identified as women. The first author (LI) facilitated the overall project and all meetings, encouraging participants to share experiences, reflect on their own and others’ experiences, and work towards agreement. The facilitator identifies as a woman and is a nurse practitioner at the study hospital, but not in the ICU. All participants provided informed consent and were given a gift voucher to offset any costs associated with participation.

### Co-design methods and virtual adaptations

Our engagement activities targeted foundational co-design activities (defining roles and responsibilities) through to design, implementation, data collection and evaluation. Although not proposed as a census of all recommendations or steps to follow when conducting co-design research, we used the synthesized methods by Slattery et al. [1] to help to organize a framework for participant engagement. These methods include investing in co-design, performing a needs assessment, visioning roles, responsibilities, and rewards for participants, validating participants, organizing each interaction carefully, leading the engagement, valuing participant time and input, and evaluating and reporting findings.

Since large group in-person meetings were restricted during pandemic conditions, wherever possible, we translated these methods to exclusively virtual modes of activity. Preparatory video links using a dedicated YouTube channel were shared by email to provide participants with background information and prepare them to participate in the design stages. Other virtual adaptations included use of videoconferencing software (i.e., Zoom) for all synchronous and sub-committee design meetings (Table 1). The chat function was disabled to prevent multiple tracks of conversation. Sub-committees were created in an ad hoc fashion to allow smaller groups to advance discrete aspects of the communication intervention. Adaptations also included using email exclusively instead of in person discussions or presentation to disseminate information about the project and co-design methods to participants.

**Table 1:** Co-design methods used with virtual adaptations

Co-design methods (1)	Methods used and virtual adaptations
*Invest in co-design*	
Allocate sufficient time and resources pay or reward participants for their time Provide training if needed	Participant stipend—email gift card Preparatory training and background information using email and video links
*Needs assessment*	
Determine project co-design needs including why how and on what will co-design participants and researchers collaborate	Shared results of qualitative research study identifying stakeholder needs including a locally tailored intervention in videos (33)
*Vision roles responsibilities and rewards*	
Set clear rules and responsibilities of all participants in co-design Clarify how co-design participant feedback will be used Ensure all parties understand the importance of co-design and the potential benefits Manage expectations and make sure that there is a shared vision and goal	Preparatory email and virtual meeting to discuss co-design ground rules, methods, participant responsibilities. potential benefits, shared vision, and goals
*Validate participants*	
Empower and nurture participants so they are confident enough to engage with researchers and the research process	Round table format during virtual meetings to include all participants Participants asked to provide feedback about perceived fair engagement using anonymous email survey
*Organize interaction carefully*	
Make sure that the meeting place is accessible and familiar Make sure that any interactions are well structured and regular Regularly communicate and update all parties prepare aids such as glossary’s images and plans as meeting facilitators Have backup co-design participants as some may drop out	Video platform for home or work office location Encouraged maximum participation using post meeting emails and asynchronous meeting methods Structured meeting electronic agendas and template for slide deck
*Lead the engagement*	
Carefully define and control the scope of the engagement Don’t let groups dominate conversations and decision-making Discussing defuse tensions	Clear electronic agenda shared prior to each meeting using email Round table discussion format to prevent domination of discussion by any group or participant Acknowledgment of and diffusion of tensions through discussion
*Value participant time and input*	
Build trust and rapport between researchers and co-design participants Give co-design participants some choice and control	Members were thanked and celebrated for participation and feedback every week during virtual meetings and by email Elements of intervention and implemented selected by co-design committee
*Evaluate and report*	
Document all engagement processes Evaluate processes and outcomes based on predetermined criteria Report findings	Tracked discussions and decision points Reported evaluation and findings

Across the five co-design meetings, the percent of participant attendance varied between 55 and 82%. Easy, Attractive, Social and Timely (EAST) principles were integrated to enhance the flexible virtual approach to participation. This included the planned use of synchronous, asynchronous, and sub-committee meetings (Table 2) [12]. To promote maximal committee participation, after each design meeting, a summary was sent by email to all participants (i.e., those who attended and those who did not) with links to electronic surveys (i.e., Survey Monkey) for anonymous voting on key decision points. Synchronous co-design meetings had an attractive PowerPoint slide layout as well as structured (i.e., templated presentation with summary and discussion questions, evaluation) and unstructured facilitated components (i.e., open, and round table discussion). All templates and communications were branded with the project title “COPE”.

**Table 2:** Application of EAST (12)  principles

EAST principles	Application
*Easy*	
Use defaults Reduce hassle Simplify messages	Pre-set meeting dates established Asynchronous methods to increase participation Three stages (preparation, intervention design, implementation plan design)
*Attractive*	
Attract attention Rewards	Co-design posters on unit Attractive templates for all meetings and communications Organization leadership celebrated participants’ contributions Provide stipend Professional rewards for participation (e.g., curriculum vitae, participation in research poster development)
*Social*	
Show that participation is desirable Networks Encourage commitment	Reviewed importance of and power issues in co-design with participants Participant group cohesion through establishment of rapport
*Timely*	
Prompt when most receptive Consider costs/benefits Help people plan response	Planned timing with unit leadership to ensure no conflicts with other projects Considered time constraints of participants in timing and length of meetings and participation in feedback provision (e.g., post meeting immediate feedback and short evaluations)

Several strategies were used to ensure maximum participation in co-design. Planned asynchronous sessions were conducted in response to anticipated limited participant member availability due to time constraints for staff in the ICU. Asynchronous sessions included the email distribution of a prepared summary presentation with attached discussion questions. Using pre-set meeting times and dates, timing the co-design activities to not conflict with other unit projects, having a consistent and clear message, celebrating participation with messages of thanks, establishing participant rapport through pre- and post- meeting conversations using video platforms, leadership awareness and endorsement of co-design activities through email messages, participant stipends, and the use of clear goal-oriented electronic agendas were intentional attributes of the co-design methods used to improve participant engagement. Finally, sub-committee work was integrated using small group Zoom sessions to focus on specific areas of intervention and implementation design, with reporting back to the large group.

### Data collection

At the end of each virtual co-design meeting, we conducted a brief anonymous emailed evaluation of participant understanding of and experience with co-design methods. The evaluation comprised a four-item questionnaire about participant experience during the co-design using a yes/no response frame and space for narrative feedback. All co-design meetings were audio and video recorded. Data collected included virtual meeting transcripts and field notes (i.e., facilitator notes including key decision points). The facilitator also kept reflexive meeting notes documenting areas of agreement or disagreement among participants, and with her own presuppositions. Recordings were deleted immediately after taking notes. Verbatim transcription was not conducted, and no identifying information was transcribed.

### Data analysis

We summarized demographic information and questionnaire responses including proportions of affirmative responses. Verbatim qualitative responses from narrative feedback about benefits and challenges of participating in this virtual co-design project were reviewed to establish exemplar quotes. Since the survey was conducted anonymously, reported quotes do not include participant information.

## Results

### Participant experience

All (100%) participants confirmed they felt the virtually adapted co-design strategies were used as presented to them in the preparatory stage, understood the rationale for using co-design, and felt that their input was valued and validated in all interactions (Table 3).

**Table 3:** Participant survey results

Recommendation	Survey Question	Results (%Y)
Invest in co-design	Were the principles of co-design explained to me?	100
Needs assessment	Do you understand how and why co-design is used in this project?	100
Validate participants	I had the opportunity to provide input at each meeting?	100
Value patient time and input	The overall tone of the meetings was respectful?	100

### Benefits and challenges

Benefits of participation in this virtual co-design methods included an appreciation for the attention to diverse and equitable views from the committee. Participants were energized by “the exchange of ideas” and felt the process aligned with their professional interests and obligations in “understanding the patient experience”. Participants reported that they developed a better understanding of the roles of their colleagues and appreciation for the strengths of multi professional collaboration, and a sense of group cohesion that transcended the virtual meeting space. One participant wrote:“… [I enjoyed the] opportunity to understand the perspectives of each stakeholder at each step of the co-design process.”

Virtual methods and flexible meeting approaches using EAST principles allowed for opportunities for input during meeting absences due to unpredictable pandemic work schedules. One participant wrote: “Everyone had a chance to provide input which was evident during the discussions following [each] meeting.”

Challenges to the virtual co-design process included limitations of virtual methods for hands on design of the intervention prototype. Videoconferencing as a method for meeting collaboratively, though necessary during COVID-19 IPAC conditions, was considered not ideal for creation of a physical cart placed in the ICU to house a variety of communication tools. One member wrote: “[I would have preferred] meeting in person to actually review the tangible ingredients to the communication cart. It would have been lovely to meet in person to touch/feel the cart items and discuss the functionality of use.” In person meetings were perceived to more easily advance working relationships, brainstorming sessions, decision-making, and understanding of material intervention elements such as tools.

Another reported challenge to the co-design methods in general were that “as always, getting a group of people together” can be challenging in the busy hospital setting which was exacerbated by the pandemic. Despite employing EAST strategies to improve participation, unpredictable work schedules inhibited consistent attendance by some participants.

## Discussion

The current paper reports on the co-design methods, benefits and challenges of adaptation from face-to-face engagement to a virtual delivery mode and application in the production of a communication intervention for ICU patients during the COVID-19 pandemic. Adaptations included distribution of foundational video education for co-design participation, electronic meeting agendas, email meeting summaries, evaluations to seek participant experiences and feedback, electronic voting on design elements irrespective of meeting attendance, and a flexible meeting plan. Other key adaptations included the use of email for distributing information, videos, and electronic evaluations as well as the use of a videoconferencing platform for synchronous meetings. Positive participation experiences and some challenges included incorporation of diverse viewpoints in the design, but a lack of hands-on time and participant availability to meet regularly.

Virtual adaptations of recommended co-design methods described in this paper include the use email, videoconferencing platforms, and other electronic based tools that were both free and easy to disseminate. Though contextually distinct, the virtual adaptations described in this paper align with those in the extant literature [9–11, 25]. Fails et al. [9] used the Zoom platform for co-design meetings, including the chat and breakout room features. Fails et al. [9] used electronic whiteboards for prototype design which is a potential creative solution to the “lack of hands-on” barrier reported by participants in this study. Kennedy et al. [10] also described using Zoom for meetings, as well as breakout rooms and polls to facilitate intervention design. As with our study, these authors adapted face-to-face intervention design meetings with additional methods to build relationships, share and discuss design ideas, and maximize participation.

Use of co-design methods for intervention design in the ICU are not commonly reported, contributing to barriers to uptake of evidence to practice and negative outcomes for patients, family, and HCPs [23]. Pandemic conditions in the ICU exacerbated stress and time constraints for staff [14]. However, previous research in this setting revealed a desire by HCPs to be included in practice decisions and intervention design [14]. Without comparable data we felt participant attendance rates were an expected and reasonable outcome for co-design in ICU during a pandemic. The multimethod virtual approach we used, included multiple layers and opportunities of participating and collaborating such as a pre-planned combination of electronically disseminated videos, video meetings, brief email summaries, and small (1:1) meetings. Campbell-Yeo et al. [11] described the use of virtually adapted co-design to create clinical pathways in the neonatal ICU in what they described as an ‘agile’ collaborative process including co-design sessions and live virtual document review. In the ICU context, using virtual adaptations of co-design methods and other methods of information and idea sharing is both practical for difficult to reach clinicians and maximizes equity concerns for informing intervention design for impaired ICU survivors.

Use of virtually adapted methods permitted the inclusion of patient survivors and family from home without the need for travel. Recovering from critical illness is often accompanied by a high burden of morbidity for both patients and family [26, 27]. Travel to study sites for face-to-face design meetings makes participation in co-design projects for ICU patient survivors and family difficult [26, 27]. Fails et al. [9] and Kennedy et al. [10] also report virtual connections permitted inclusion of participants across geographically remote areas. Kennedy et al. [10] further report potential cost savings associated with using virtual co-design methods versus traditional face-to-face methods since participants need not travel nor be reimbursed by researchers for transportation or childcare. Participant socioeconomic status (i.e., need for computer and intranet access) should be addressed in the design to ensure equity to participation.

Participants in this study reported positive feelings and insights into the experiences of colleagues, patients, and family. In retrospect it is impossible to tease apart the reported positive feelings and benefits of participating in co-design, from using virtual methods. That said, the employment of virtual methods to facilitate a co-design, which the participants reportedly appreciated. In co-design, careful facilitation allows for disagreements stemming from professional or personal experiences to be aired and critically discussed, resulting in transformational learning rather than mere consensus [28]. In addition to being feasible during pandemic conditions, engagement with willing HCPs in a co-design project may have been a positive experience among the abundant negative psychological experiences of ICU HCPs reported during the COVID-19 pandemic [29].

Despite successfully adapting to virtual methods and numerous benefits, participants also reported challenges to using co-design in this project. Participants acknowledged the flexibility in methods to participate but were still attuned to their own and other’ difficulty attending all meetings, a challenge reported by other researchers conducing co-design in critical care environments during the pandemic [11]. Previously reported challenges to co-design in healthcare generally have included increased time, cost, tensions between researchers and non-researchers in decision-making, and concerns related to issues of power [1]. Specific to using virtually adapted methods, Fails et al. [9] add reported challenges such as technical glitches, background conversations in the chat box, distractions, and multi-tasking, and Zoom fatigue interfering with on screen participation. [1, 3, 30]. Mitigation of these challenges were attempted using multiple methods for member participation, setting a relatively short work timeline for design, and using published recommendations for setting expectations and norms for co-design participants [1].

### Strengths and limitations

Strengths of the design include detailed reporting of methods used and evaluation of recommended co-design processes adapted to a virtual environment. The development of virtual co-design methods is an important additional research method for anyone (researchers or practitioners) wishing to work with users not only during a pandemic but also when needing to overcome geographic limits or operating with limited resources (e.g., low budget). Multiple means of participation (e.g., synchronous and asynchronous) for participants add to the strengths of this design, ensuring diverse views were presented and integrated in the final design.

There are also limitations. Despite efforts to maximize participation of participants in every element of the design and the need to keep to a tight timeline, the course of intervention design was relatively short. We aimed to have more variety in the professions represented, however, results might be biased in favour of participants and professions who have a particular interest and experience with communication support (i.e., SLP, asymmetric gender representation) and may not represent the views of the entire ICU. Additional time to trial and revise the intervention might have had design implications for end-user acceptability. There were also limitations in recruitment of patient survivors and family, attributable in part to the ongoing pandemic and high morbidity and mortality of patients in the study site, particularly during a pandemic. Finally, due to the small number of participants in our co-design committee, although surveys were anonymized, participants might not have felt this was possible and this might have influenced their responses. Clinician participants also knew the facilitator professionally, and although efforts were made to encourage open and honest responses, their responses might have been influenced by this prior relationship.

## Conclusion

This report describes the methods, benefits, and challenges of adapting in-person co-design methods to a virtual environment to produce a bundled communication intervention for use in the adult ICU during the COVID-19 pandemic. Co-design methods contributed to positive participant experiences and a rapid design outcome. The reported methods demonstrate a feasible, practical, rapid, and low-cost approach to intervention virtually adapted co-design suitable to pandemic and non-pandemic healthcare context. Adapting co-design methods to a virtual approach also functioned to fulfil recommendations and produce design elements that built on knowledge of local barriers and facilitators towards innovation.

### Supplementary Information

**Additional file 1.** Intervention components

**Additional file 2.** Elements of the final implementation plan

## Data Availability

All data generated or analysed during this study are included in this published article [and its supplementary information files].

## Abbreviations

ICU: Intensive care unit
HCPs: Healthcare providers
COPE: Co-designed bundled patient communication intervention for use in the ICU during and beyond the COVID-19 pandemic
IPAC: Infection prevention and control
EAST: Easy, attractive, social, and timely
PPE: Personal protective equipment
