# Supplementary material for: Adapting co-design methodology to a virtual environment: co-designing a communication intervention for adult patients in critical care
Source: Res Involv Engagem. 2023 Nov 13;9:103. doi: 10.1186/s40900-023-00514-6 (PMC10644625; doi:10.1186/s40900-023-00514-6)
Supplement: Supplementary file 1 — Additional file 1. Intervention components [file 40900_2023_514_MOESM1_ESM.docx]

Additional file 1: Intervention Components

The COPE intervention and implementation plan is reported below using TIDieR Checklist template (34).

1. Brief Name

Co-designed bundled COmmunication intervention for adult ICU patients with an advanced airway during the COVID-19 PandEmic (COPE).

1. Why

COPE is a co-designed bundled communication for use in the adult ICU with patients who have an advanced airway. It has been designed to be used during and beyond the COVID-19 pandemic, when infection prevention and control measures are in place.

Co-design was used as part of MRC guidance for complex intervention design to ensure the intervention matched the needs of end-users. A bundled approach was used to ensure the intervention addressed the variety of barriers identified in the evidence-base as well as during the exploration of barriers during Phase 1 of this study.

1. What

Materials: The COPE intervention has three main components:

1a. Communication cart:

- - - Drawered cart
    - Located centrally in the ICU
    - Stocked with evidence-based communication tools

Board packages included pointing boards:

- - - - Alphabet
      - Needs – picture
      - Pain scale
      - Blank 8 box

And assessment tools:

- - - - Communication assessment
      - Communication treatment algorithm

Other tools

- Pencils
- Wipeable clipboards
- Loose paper
- Coil books

1b. Virtual tool cart

- Located on site intranet critical care page
- Printable PDF communication tools (as above)
- Links to the SPEACS-2 website, and Patient Provider Communication websites for additional resources including translated communication tools (35, 36)

2. Patient communication skill education

- In-person brief training at huddles and walk-arounds
- Links to the SPEACS-2 training modules

3. Family connection

- - Up to date information about online visiting – link to institutional visiting policy, virtual visiting instructions

1. Procedures

The ICU team was oriented over 8 weeks to the components of the carts. Both carts were accessible to the ICU team 24/7. Training modules were made available but were not mandatory components of the implementation. Training topics were taught during unit-wide huddles.

1. Who

Internal and external facilitators were used to implement the intervention.

Internal facilitator: Speech and language therapist for ICU, Masters prepared with over 10 years ICU experience. Extensive education on training patients, family, and HCPs with communication difficulty.

External facilitator: PI. Nurse practitioner for unit adjacent to ICU. Masters prepared with previous ICU experience of over 5 years.

Leadership: Unit and organization leadership were invited to attend huddles to encourage staff to continue efforts supporting patient communication.

Target audience: all ICU HCPs (nurses, interprofessional team, leadership) and ward clerk.

1. How

Intervention was introduced over 8 weeks. Modes of delivery included:

1. Email: Introducing the program to the ICU email list (all current HCPs).
2. In-person: The facilitators were at the study site 3 days/week during the implementation. They attended and presented communication skills at daily huddles and did walk-arounds to review the skills. During walk-arounds the facilitators reviewed the contents of the carts and did case-based reviews using the communication treatment algorithm to assist front-line staff in assessing patients and selecting appropriate communication tools.

Awareness building of speech and language therapist role in ICU with regards to communication support was also incorporated into the in-person delivery.

1. Posters and signs: A consistent design for wall poster and swag items marketing the program were used. Items included pens, t-shirts, and retractable lanyards with the word “COPE” on them.
2. Where

In the study setting.

1. When and how much

3 days/week for 8 weeks from 0800 – 1700.

1. Tailoring

The implementation was tailored to the local site during the co-design phase (Phase 2).

Key recommendations included:

- - Use of huddles
  - Multi-modal education
  - Non-mandatory e-modules
  - Minimal wall posters and use of alternate signage (e.g., pens)
  - Review by infection prevention and control team to ensure tools are appropriate
  - Include an instruction page for cleaning/disposal
  - Invitation of leadership to celebrate
  - Use of patient stories to illustrate importance and use of communication interventions in the ICU

1. Modifications

Modifications included:

- Use of 1:1 training of interprofessional team who did not regularly attend huddle
- Extension of implementation to 8 weeks from original plan of 6 week
- Addition of instruction page for cleaning/disposing of tools placed on the communication cart
- Less emphasis on Family Connection component since not as acutely needed in between COVID-19 waves

1. How well planned

- The PI monitored implementation activities and tool use/re-stock on a weekly basis.

1. How well actual

- The implementation ran as intended for 8 weeks, 3 days/week.
- The cart was stocked on a weekly basis
- Modifications are described above
