# Supplementary material for: Adapting co-design methodology to a virtual environment: co-designing a communication intervention for adult patients in critical care
Source: Res Involv Engagem. 2023 Nov 13;9:103. doi: 10.1186/s40900-023-00514-6 (PMC10644625; doi:10.1186/s40900-023-00514-6)
Supplement: Supplementary file 2 — Additional file 2. Elements of the final implementation plan [file 40900_2023_514_MOESM2_ESM.docx]

Additional file 2: Elements of the Final Implementation Plan

| Key components | Activities |
| --- | --- |
| Co-facilitation | - External and internal facilitators - Use of implementation science and best evidence |
| Huddle | - ICU huddle every Monday and Wednesday - Invite leadership to huddle - Introduce bundle components and brief teaching |
| Email | - Introduce bundle components - Link to communication web page |
| Involve stakeholders | - Describe co-design and involvement of stakeholder feedback - Team members story telling of communication experiences at huddle |
| Posters/Give aways | - Give aways with logo (e.g., pens, lanyards) - Raise awareness with a raffle - Prizes for participation at huddle (e.g., t-shirts, treats) |
| Bedside teaching/walk abouts | - Every Monday and Wednesday after huddle - Bedside review of components - Elicit and integrate real-time feedback |
| Include leadership | - Leadership at huddle - Celebrate dedication to excellence in patient care |
| Audit/Feedback | - Cart usage - Audit of implementation activities |
